# Wireless Network for Measurement of Whole-Body Vibration

**DOI:** 10.3390/s8053067

**Published:** 2008-05-06

**Authors:** Diogo Koenig, Marilda S. Chiaramonte, Alexandre Balbinot

**Affiliations:** 1 Federal University of ABC – UFABC, Programa de Pós-Graduação em Engenharia da Informação Rua Santa Adélia, 166 – Bairro Bangu – Zip Code: 09.210-170, Santo André, SP, Brazil; E-mails: abalbinot@gmail.com; aulaeng@gmail.com; 2 University of Caxias do Sul – UCS, Electrical Engineering Department – NPEngBio Alameda João DalSasso, 800 – Zip Code: 95700-000, Bento Gonçalves, RS, Brazil; E-mail: marilda.chiaramonte@gmail.com

**Keywords:** ZigBee, wireless network, whole-body vibration, acceleration

## Abstract

This article presents the development of a system integrated to a ZigBee network to measure whole-body vibration. The developed system allows distinguishing human vibrations of almost 400Hz in three axes with acceleration of almost 50g. The tests conducted in the study ensured the correct functioning of the system for the project's purpose.

## 1. Introduction

With the evolution of technology new standards and communication systems are coming into the new markets. Wireless networks (Wi-Fi, Bluetooth, ZigBee, among others) are even more present in people's daily life. In many experimental situations it is interesting to point out the reduction of cables that connect the transducer to the conditioning system, mainly because of the distance involved, the difficult access to the measure points, the noise, among other factors [1-2].

Many acquisition systems, for occupational vibrations use many cables, point to point, big and medium size and they don't allow simultaneous integration of many sensors [2-15]. Telemetry systems can be attached to the transducer or to the data acquisition systems, allowing the transmission and reception of the interest signal with the reduced use of cables. In general, a wireless telemetry system can be composed by sensor, signal conditioning, multiplexers, analog-to-digital converts (ADC), microcontroller, power supplies, data communication and storage systems. As for wireless sensor network, many network topologies (star, mesh, etc) and standards are found [2].

The study of human vibration was selected because exposition to regular vibration may contribute to the appearing of musculoskeletal disturbances in professionals [2-6]. Besides, regarding the development of manual machines and projects for vehicles (especially in seats and suspension systems) the study of vibration in humans has the objective of minimizing the effects to health and comfort [3-14].

Therefore, this study presents a system of portable telemetry using the ZigBee network for acquisition, with simultaneous sampling and data storage aimed at the occupational vibration characterization in order to evaluate the exposition levels of the accelerations to which the human body is submitted when the vehicle operates in a plane and flat terrain, simulated by a vibratory platform.

## 2. Human Vibration

Researches [3-14] showed that vibration is one of the common occupational risks. The increasing exposition to the human body vibration, in workers who operate tractors, digging machines and too many vehicles is incomplete, however there are evidences that regular exposition to the vibration can contribute to the generation of back pains in professionals such as bus drivers, tractor operators and helicopter pilots.

According to Griffin [3, 6], the exposition to the human body vibration is related to problems in the muscular/postural activity, circulatory system and the appearance of intramuscular disturbances. In that sense, with the increasing use of vehicles and of new kinds of seats, investigation of the acceleration levels and of necessary comfort is carried out as well as its dynamic characterization. Using the vibratory platform prototype for seats (shaker for seats), it is possible to characterize the vertical accelerations level to which a driver is submitted.

Epidemic studies such as the one carried out by Johanning *et al.* [4] showed the high rate of problems on the back of operators in the subways of New York City. They concluded that the great discomfort and the back pains can be related to poor ergonomics of the seats and the high exposition to the vibration by subways, Hoy *et al.* [5] developed a study to verify the vibration levels in trucks, because according to the authors, in Europe, from 10 to 15% of all illnesses are related to pains in the back and their study determined that there is a significant relationship between vibration and these pains. Tripepi *et al.* [6] registered those vibrations, in the frequency band from 2 to 80Hz, are transmitted to the human body as a potential source of risk to the pilots' health by locomotives. Hulshof *et al.* [7] evaluated several European health services regarding the vibration and the back pains. They verified that the vibration is considered an occupational disease and that a few countries have established limits for the exposition.

The human body can be considered as a sophisticated biomechanical structure and the sensitivity to vibration can involve several factors, such as, posture, muscular tension, frequency, vibration amplitude and direction, and In addition to that the duration and dose of the exposition [3, 9].

### 2.1. Measuring of Human Vibration

ISO 2631 [8] presents methods for the vibration quantification in the human body regarding human health and comfort. It can be used to evaluate vibrations generated by vehicles (air, land and water) and machines where people are exposed to the mechanical vibrations that can interfere in comfort, in occupational activities and in human health: (a) vibration is measured according to a system of coordinates originated in a point in which the vibration is entering the body through each one of the axes; (b) transducers should be located to indicate the vibration in the interface between human body and the vibration source, or the nearest possible to a point or area referenced; (c) the parameter for vibration magnitude evaluation is the acceleration rms (root mean square) that should be expressed in *^m^*/_*s*^2^_; (d) the dynamic response of the human body, like that of any mechanical system, is dependent on its frequency of vibration. Human exposure to vibrations is most frequently quantified using the “frequency weighting” procedure takes into account all frequencies in the spectrum and yields similar values irrespective of which proportional-bandwidth filter is used or whether constant-bandwidth analysis is employed. Methods of obtaining frequency-weighted values from proportional-bandwidth and constant-bandwidth frequency analysis are presented in ISO guidelines [8].

The vibration amplitude is obtained through the acceleration rms given by Equation (1):

(1) $$a_{\text{rms}}=\sqrt{\frac{1}{T}\int_{0}^{T}a^{2}(t)dt}$$

where *a_rms_* is the acceleration rms it expresses in *^m^*/_*s*^2^_, *a*(*t*) is the acceleration that varies in time *t* and the integration period is accomplished for a time *T* (measure duration in seconds). The electric signal that characterizes the vibration should be conditioned, amplified, filtered (with low band filter and anti-aliasing filter with frequency cut frequency of sampling) and conversion with an ADC (Analog-to-Digital Converter) with enough resolution (typically 12 or 16 bits). The acquisition system should be able to register a frequency sample up to 300Hz, for measure channel, for the vibrations study in the human body.

### 2.2. Seating Dynamics

Several companies have developed projects related to the area of work of vehicles regarding users' comfort and health (mainly related to the ergonomic aspect), but there are few projects that relate vibration to comfort. The exposure to vibration at different frequencies or different axes can produce different feelings in different parts of the body [3-9].

When the vibration amplitude increases, discomfort usually increases, but the transmission of vibration to a person who is sitting can be significantly affected by the seats, and it therefore deserves special attention. Rowland, Johnston, Moseley and Griffin presented some studies showing that the vertical transmission of vibration to the head of a sitting person can be influenced by the backrest of the seats [3-9]. The presence of a backrest vibrating vertically with the seat can often increase the magnitude of vibration onto the head, and the changes in the body posture, in relation to the backrest, can change the transmissibility of the body [3].

It is important to consider that a very hard seat causes excessive pressure onto the pelvis and sacrum, but a very soft seat distributes excessive pressure around the hip. Many occupants of the seats of the vehicles are interested in reducing the vibration. A seat provided with optimal dynamic properties is necessary to minimize unwanted vibration to the occupants.

Thus, three factors combine to determine the dynamic efficiency of a seat: environment, seat dynamic response and human body response [3, 8-9]. The most direct method to characterize the vibration in the seats is to compare the acceleration in the seat and on its base.

## 3. ZigBee Network

The main characteristics of ZigBee network [15] are low power consumption, simple implementation, low cost interface, simplicity in the configuration, redundancy of devices, high node density per physical layer (PHY) and medium access control layer (MAC), and they allow the network to work with a great number of active devices – critical and interesting attribute for applications with sensors.

**Figure 1.**
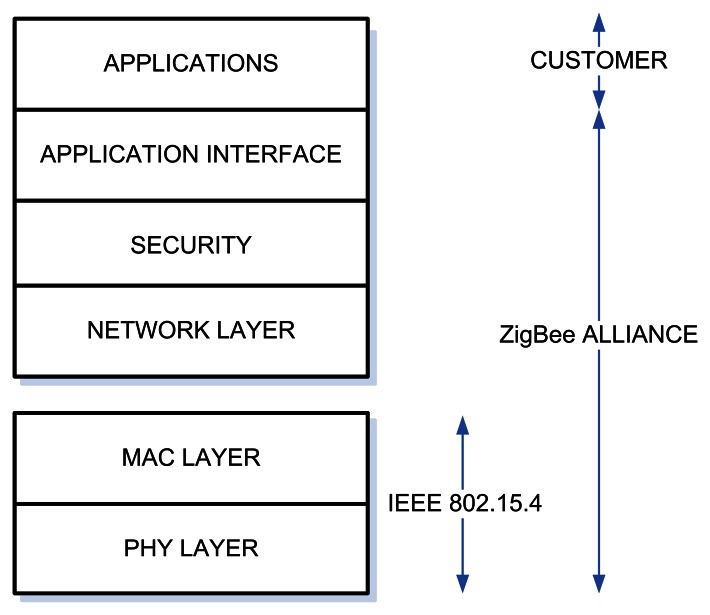
ZigBee stack.

The main applications are in predial automation, telemedicine and entertainment. ZigBee is based in the IEEE 802.15.4 [15] standard in terms of the PHY and MAC layers. The ZigBee Alliance, ZigBee's owner, added the Application Interface (API), Network (NWK) and the Security (SEC) layers to the IEEE 802.15.4 specifications to complete what is called ZigBee stack, schematically represented in Figure 1.

The PHY layer was designed to accommodate the low cost interfaces. The MAC layer allows a Reduced Function Device (RFD) to operate in the network without the necessity of great of memory capacity and also controls a great number of devices with no need to use standby mode. The NWK layer may manage high quantities of network nodes with quite low latencies [15]. The IEEE 802.15.4 ZigBee defines three operation bands: 2.4 GHz (bit rate 250kb/s – e.g., worldwide), 915MHz (bit rate 40kb/s – e.g., North America) and 868 MHz (bit rate 20kb/s – e.g., Europe) which offers 16 channels, 10 channels and 1 channel respectively.

### 3.1. Devices, network topologies and address

The IEEE 802.15.4 defines two kinds of devices: the Full Function Device (FFD) and the Reduced Function Device (RFD) [15]. The FFD has the function to coordinate the network and, consequently, has access to all other devices. The RFD is limited to a star topology configuration, not being able to work as a network coordinator, so it does not have all the protocol services.

**Figure 2.**
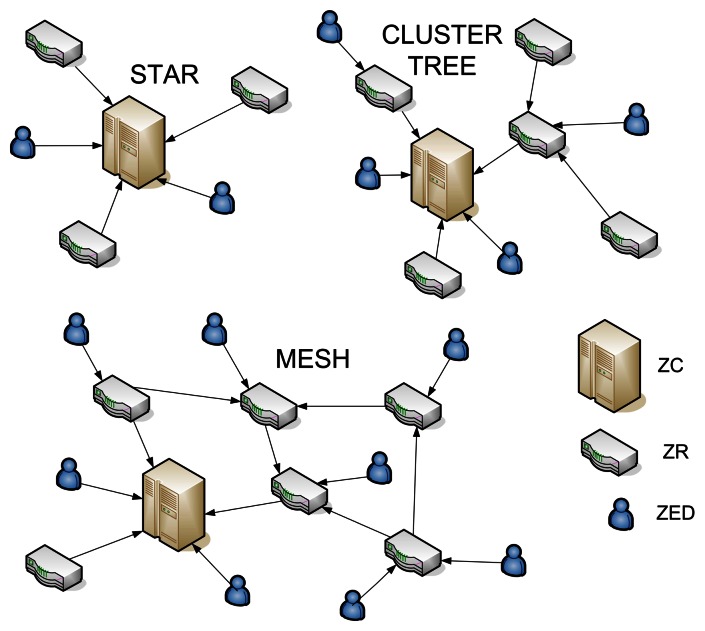
ZigBee network topologies: star, cluster tree and mesh.

The FFD and RFD devices, defined by IEEE 802.15.4, can operate in three different ways at the ZigBee standard: as the ZigBee coordinator (ZC), ZigBee Router (ZR) or ZigBee End Device (ZED). The main functions of the ZC in the network (obligatorily an FFD device operating in active state) are parameter adjustments, information transmission, nodes management and information distribution between the nodes. The ZR (obligatorily an FFD) acts as an intermediary router, sending data to other devices, preserving the local network, keeping contact with its nearest neighbors, storing and transmitting data which are of interest of its associate devices (also known as child). The ZED can be of a RFD or FFD type not having the responsibility of routing network. They join the network through routers or coordinators, also known as parents. The NWK layer supports three topologies: star, cluster tree and mesh, according to Figure 2.

A star topology consists of a coordinating node and one or more final devices (FFD or RFD) which communicate with the ZC. At the cluster tree the final devices can be associated to the network by the ZC and the ZR which have two functions: increase the number of nodes and the network scope. At the mesh topology, the FFD can distribute messages directly to other FFD. To enter the network, each device receives an address given by ZC or a ZR. At the highest level of the network, an entity known as stack profile is defined, which is a set of parameters including the following definitions: maximum network depth, maximum number of ZR and ZED. Each ZigBee network node has two addresses: one MAC address of 64 bits (EUI – 64) and a network address of 16 bits [15]. In unicast message, the node destination address is given in the heading of the MAC layer and only the device which has the address indicated will receive the message. In a broadcast pack, the destination address at the MAC layer is default (FFF_16_) and any transceiver connected will receive a message. This addressing way is used when the device is associated to the network, determining routes or performing other functions of the ZigBee protocol [15].

### 3.2. Security and kinds of traffic

ZigBee standard adopted the safety algorithm based on the simplification of the routing algorithm Ad-hoc On-demand Distance Vector (AODV) [15]. The MAC layer uses the Advanced Encryption Standard (AES) as its encriptation algorithm. When the MAC layer transmits (or receives) a frame, it checks the destination (or the frame source) and then recuperates the key associated to this destination (or source) and then uses this key to process the frame according to the safety routine indicated for the key being used.

Each key is associated only to one safety routine and the frame MAC heading has one bit that specifies if the safety for the frame is free for use or not. The MAC layer is flexible to guarantee the traffic flow of periodical and intermittent data [15]. A ZigBee network is established through a ZC that looks for another ZC in the channels permitted. Based on the number of network found in each channel, the ZC establishes its own network with a single identification of 16 bits – called PAN ID. Having established the network, ZR and ZED may join it. ZigBee devices store information about other network nodes in the nonvolatile memory.

## 4. Experimental Section

**Figure 3.**
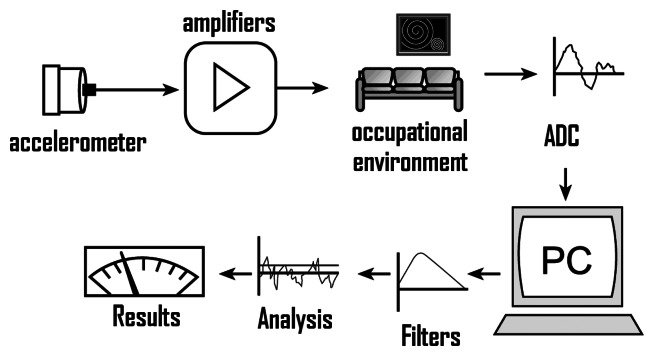
Arrangement for a typical experimental measurement of occupational vibration.

A typical system to characterize occupational vibration is found in Figure 3, where the transducer (in the example, accelerometer) is positioned to detect the vibration axis.

With appropriate conditioner the signal of accelerometer can be converted to digital signal and interfaced to a digital system to perform signals processing routine, such as filters, dispersion measures, FFT, spectral density, among other things, allowing results generating by phenomenon under study. Vibrations can be measured with transducers of displacement, velocity, acceleration and frequency.

The accelerometers are found in various sizes, technologies (piezoelectric, piezoresistive, capacitive, heat, etc) [1-2]. There are several types of packages, axes of measurement, different tracks of amplitude and frequency. The basic principle of any accelerometer is the action of acceleration in an inertial mass, coupled to a transducer, to generate force – Second Newton Law.

Piezoelectric accelerometers have high sensitivity (compared to piezoresistive) and respond to deformation lower than 1μm. They are appropriate to measure variable efforts such as force, pressure and acceleration. Their small size (may be smaller than 1mm) and the possibility of manufacturing devices with unidirectional sensitivity properties is interesting for many applications, particularly in the monitoring of vibration. Piezoresistive accelerometers are implemented with strain gage sensors in half-bridge configuration or complete-bridge Wheatstone. They are designated for low frequencies (below 1 Hz) and can be used as inclinometer, unlike piezoelectric accelerometers. The capacitive accelerometer can also be used as inclinometer and presents stable frequency response according to the temperature (gives better stability than the piezoresistive), but is not linear.

Mechanical systems usually have dynamic behavior in the frequency range from 10Hz to 1kHz, however, in the study of human vibration, the range of interest, depending on the segment under study, is between 1Hz to 300Hz (for the spine: up to 20Hz). There are several parameters that may be used to characterize a signal derived from an accelerometer [2-3]. In time domain, what outstand are the parameters derived from the analysis of amplitude, with emphasis in the peak value, variance, rms. value, peak factor, frequency and duration. In frequency domain Fourier Transform is essential, allowing the conversion of time function on a continuous basis in frequency.

### 4.1. Wireless Telemetry with ZigBee

**Figure 4.**
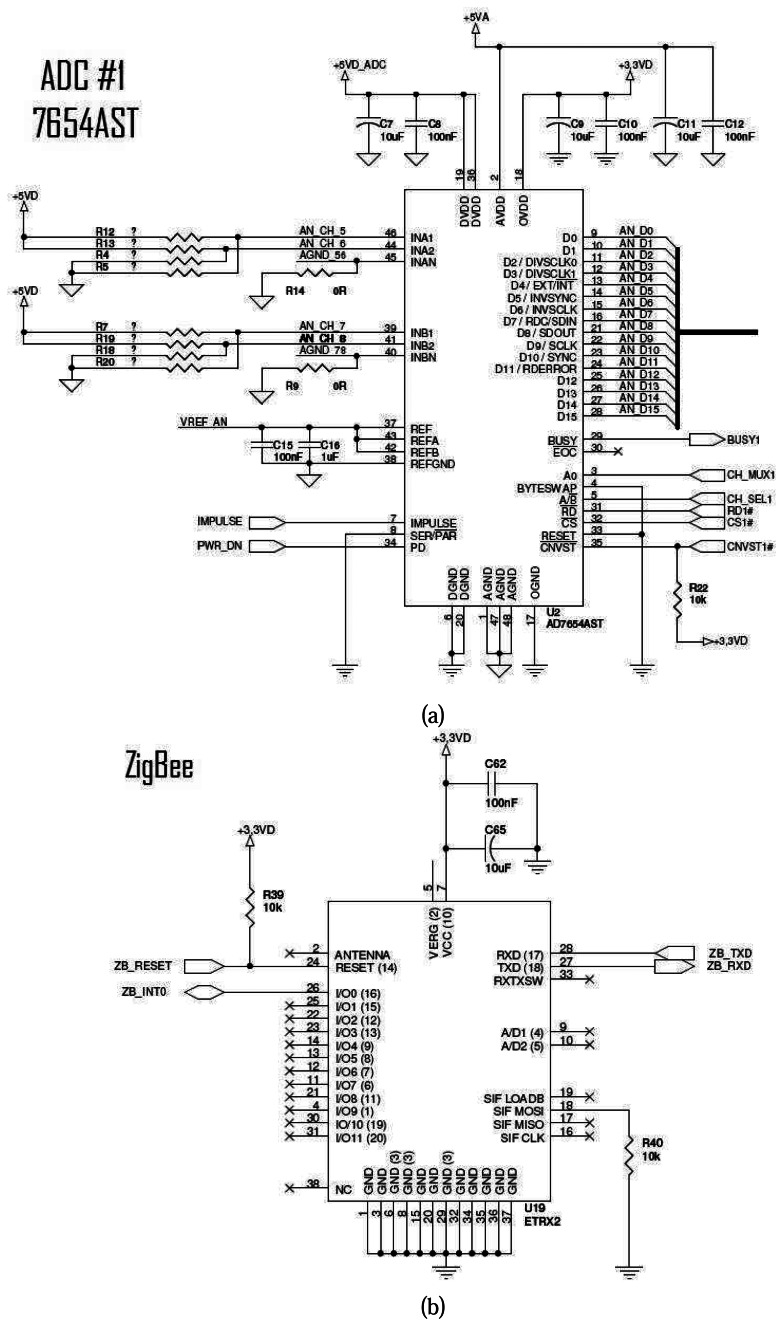
Electrical diagram: (a) ADC7654AST and (b) ZigBee.

**Figure 5.**
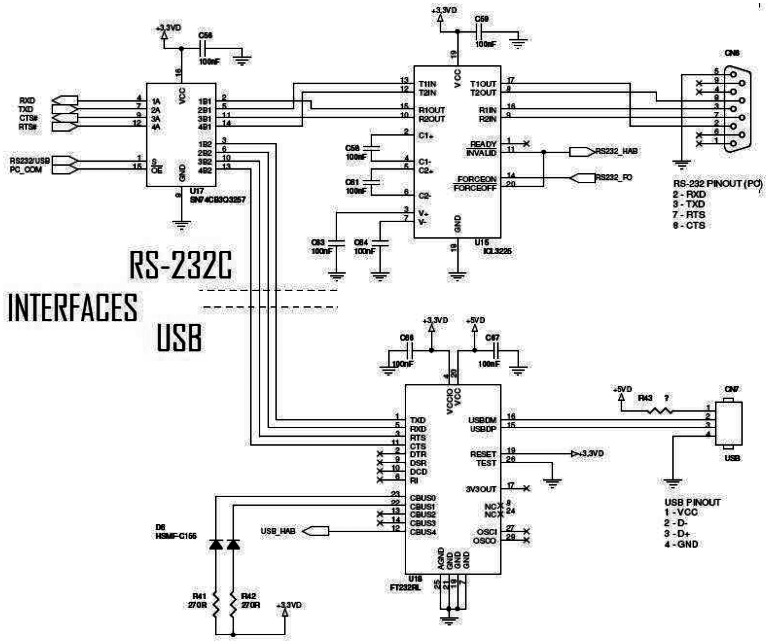
Electrical diagram: interfaces - RS-233C and USB.

**Figure 6.**
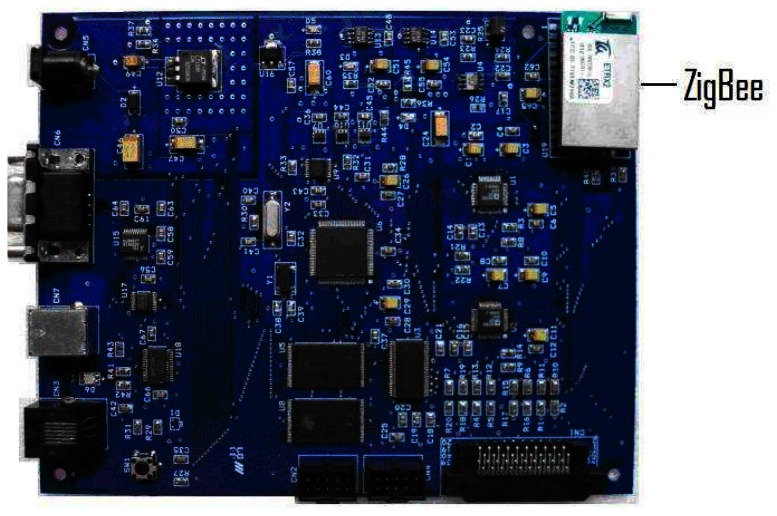
Photograph of the system developed.

The developed system is made of two boards and uses the ZigBee network to transmit and receive data. The main system features are: four layers printed circuit card, with isolated ground layers for analogic and digital circuits; reversal polarity protection; voltage pre-regulator operating from 7.5V to 28V, current consumption lower than 150mA; low noise voltage regulators from 5V and 3V isolated in analogic and digital circuits; 16-bit microcontroller operating at 32MHz (PIC24FJ128GA); RS-232 and USB communication interfaces; wireless communication ZigBee (ETRX2) interface; 4MB SRAM memory buffer for data storage of the trials, two 16-bit ADCs with four channels each (AD7654), up to four simultaneous sampling channels and 0 to 5V analog input (see Figures 4, 5 and 6– electrical diagram and photograph of the system developed).

The system performs successive acquisition of the the selected ADC channel, until the memory is completed. After that, the data are transmitted to the remote terminal by ZigBee transceiver. With this working procedure higher acquisition rates can be developed because the data are transmitted from the memory to the communication module with a slower rate. It's important to emphasize that the transducer – besides moving with the structure – can't change the dynamic characteristics of the seat or the body (there are suitable devices for this application in the market, for example, the seat pad made by Endevco Corporation used in this project).

**Figure 7.**
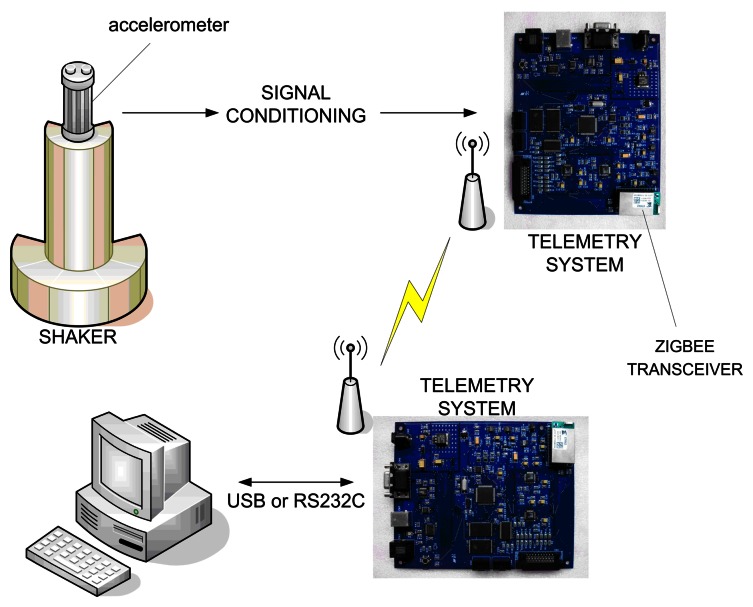
Experimental arrangement for calibration of experimental system.

In addition to that, the system must allow the acquisition, transmission and reception of acceleration in different magnitudes (up to 50g) and frequency rates (1Hz to 400Hz). Figure 7 shows the outline/draft of the gauging system, where the sensor (an accelerometer piezoelectrical model 65 of Endevco Corporation) is placed to detect the main vibration axis. The exit of voltage from the sensor varies lineally with the acceleration and is conditioned by the circuit (gain of 1000, high-pass filter with cut frequency of 0.12Hz and low-pass filter with cut frequency of 400Hz).

Then it is digitized by ADCs of the acquisition board and sent to the reception board. The receiver board is interfaced with a PC through the RS-232 or USB. In the computer, a program, developed in LabView 8.0, performs routines to signal processing, such as frequency analysis (Fourier spectra, power spectra, among others), digital frequency weighing, amplitude analysis (peak, mean, rms, skewness, kurtosis, rmq, among others) allowing the production of results of the phenomenon in this study. The shaker was used (Vertärkertechnik Berlin type 57/5) to generate sinusoidal signals of different magnitudes and frequencies taking into consideration the interest ranges in the application area, that is, the measure of human vibration until 400Hz. It is necessary to configure the system before using it. This process can be performed by the user, for example, sending by hyperterminal, a configuration file, containing the acquisition rate of each channel and the buffer memory size for the test storage, to the connected PC board. The settings are automatically forwarded to the remote acquisition board, which awaits the user command to start the tests.

### 4.2. Vibrational Platform

**Figure 8.**
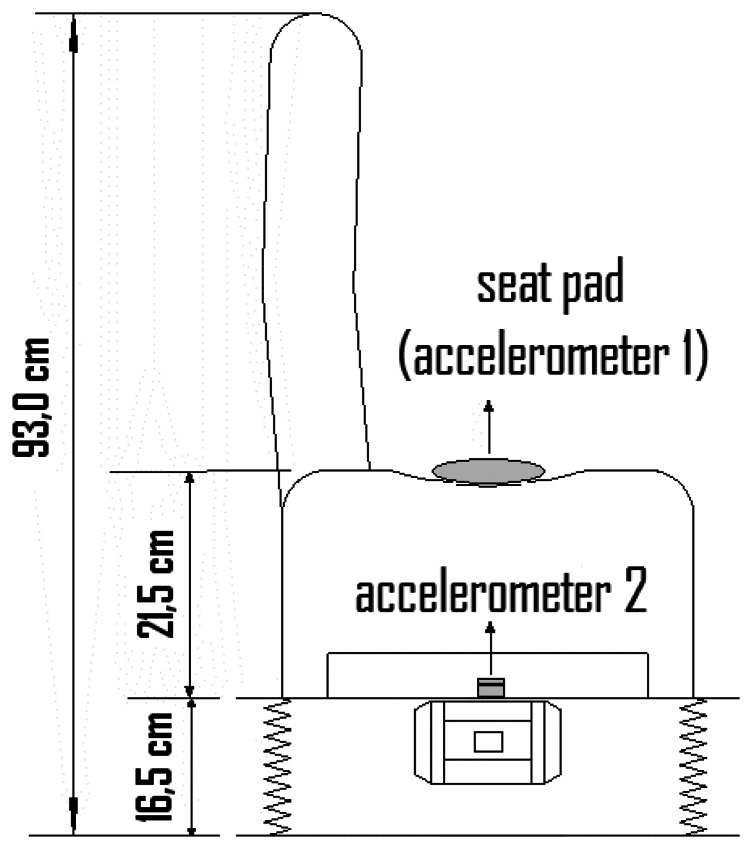
Vibrating platform outline.

To assess the dynamic behavior of vehicle seats a vibrating platform has been developed and subjected to constant sinusoidal amplitude vibration and controlled frequency range. Figure 8 shows the outline platform that was developed, whose sinusoidal vibration is generated by a three-phase asynchronous motor with non balanced sheave on its axis. The engine (WEG) has been installed on a structure suspended by four automotive springs welded into a rigid base. The motor was connected to an inverter frequency (Siemens - Model Micromaster) to control and generate the desired frequency range.

### 4.3. Experimental Procedures

The tests are carried out with the vibrating platform instead of shaker of Figure 7. The platform is set to vibrate ranging from 1 Hz to 400Hz with an accelerometer positioned in the seat (Seat Pad model by Endevco Corporation) and another accelerometer (model 65 by Endevco Corporation) positioned at the base to fix the seat onto the vibrating platform (for location of the accelerometers on the seat – see Figure 8). Both accelerometer are intertwined to the data input of the receive board, whose converters transform analog into digital and digitize these data, and then send them to the ZigBee module.

As soon as the buffer memory of the channels is full – or at the user's choice and the acquisition is interrupted - the data collected during the time of testing are forwarded to the receiving board connected to the PC. All packages are transmitted as unicasts, and wait for receipt confirmation from the remote terminal.

To increase reliability of the system, the software also makes implemented conference of packages that can be occasionally received more than once if there is a failure in the response confirmation. It's important to point out that the tests were carried out with a19-year-old, 85-kg male person, (the angle of the seat remained in 120°). The data were obtained in one day only, totaling 8 trials of 4 minutes and 8 minutes. A processing program was developed using the graphical programming language LabView [2]. In this program you can select the number of channels used in the tests, the sampling rate used and so on. The processing program is divided into two major steps: basic processing routines (FFT, rms, mean, standard deviation, etc.) and assessment outlines for vibration levels of the human body.

## 5. Results, considerations and conclusions

### 5.2. Wireless Telemetry with ZigBee

A very simple test was taken to determine the reach of ZigBee communication. Test software that alters a LED constantly while there is communication with the module was developed. The test was carried out without obstacles with the ZigBee. The receiver board was fixed 1.5 meters from the floor. The acquisition board was slowly carried apart until the LED stopped sparkling. This way, a reach distance superior to 150 meters was determined.

To assess transmitted and received signal integrity, sinusoidal signals of different magnitudes (up to 5V) and frequency (up to 400Hz) were available in acquisition board input. Analyzing the input signal and the one received by the receiver board, the perfect functioning of the system could be proved. One of the most important characteristics for any acquisition system is the systematic procedure for acquisition. For that, a reliable time base is necessary for the correct production of sampling rates with equal time between the acquisitions.

Software routines can interfere in these times, so a careful study of the development of routines and reliable validation tests were applied. The time base of the developed system is produced from a crystal of 8MHz. To guarantee more stability because of the environmental temperature variation, a low temperature coefficient crystal was selected. The load capacitor connected to the oscillator circuit has NPO temperature coefficient. The crystal frequency is multiplied by four by a PLL circuit integrated to the microcontroller and then divided by two. The internal frequency operation of the microcontroller is 16MHz, what gives a machine cycle of 62.5ns. This slight time break is the base to generate the sampling rates of the system.

The software developed uses the interruptions of the microcontroller timers to produce the sampling rates. Overall, four timers were used to produce four independent sampling rates in the acquisition of eight channels. Each time a timer bursts, an interruption in the hardware happens. However, assisting this interruption, several instructions of the software are carried out until a developed routine is effectively carried out. It is important that the latency time between the interruption and the effective execution of the developed routine is always steady, so that the sampling rate remains constant.

To guarantee steady latency time, the routines that are executed by each interruption have the same structure, accessing the same amount of variables and don't request other functions. It was also required that the software remained in stand-by, before an interruption, executing a loop that assessed a variable that changed during the interruption assistance. These latency times were assessed by an especially developed test routine. The measures reached a value of 16 machine cycles until the beginning of the first routine instruction. Finalizing the tests, the system was assessed in several sampling rates between 1 (one) sample per second and 5000 samples per second with all the channels qualified and presented correct functioning.

### 5.2. Vibrational Platform

**Figure 9.**
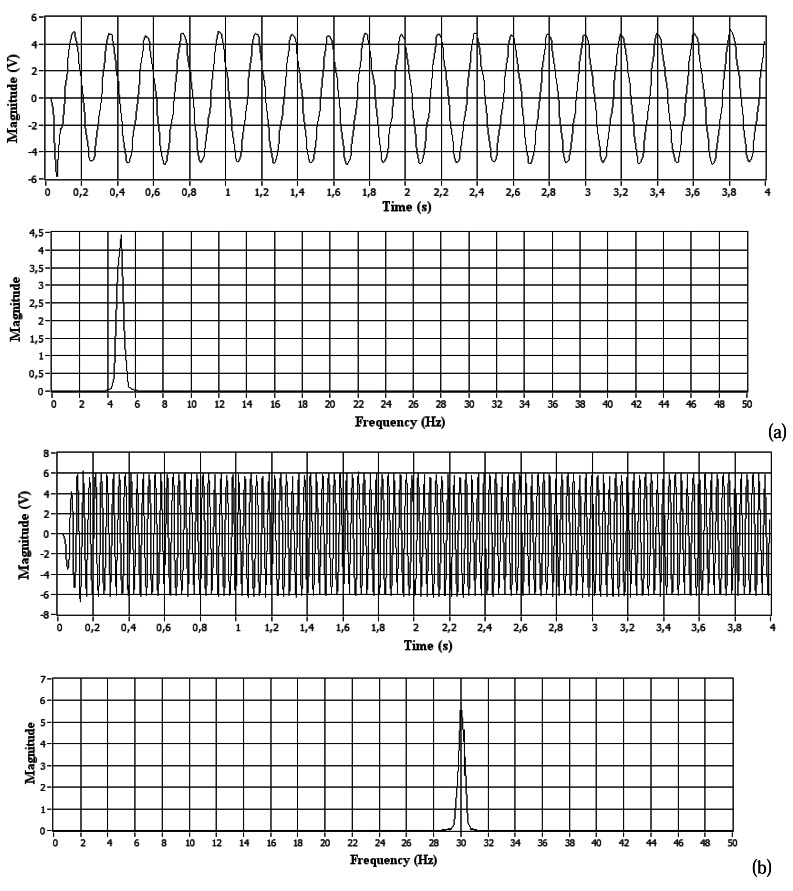
Results of the signal corresponding to accelerometer model 65: (a) 5Hz and (b) 30Hz.

Similar tests such as Figure 9 showed the proper functioning of the vibrational platform, when excited from 1Hz to 400Hz (sinusoidal signal generated by the inverter frequency). These signals were obtained on the fixing of the seat base with the platform using accelerometer model 65 (sinusoidal signal in the time domain). With mathematical processing (FFT) has been possible to verify the corresponding accelerometer sensor signal frequency (corresponds to the input signal or the excitement of the vibrational platform).

### 5.3. Human Vibration (whole-body vibration)

Table 1 shows part of the average results obtained in the test with the subject. The excitement signal is generated by the frequency inverter, and the acceleration is obtained on the base of the fixing between the seat and the platform (accelerometer model 65) and in the seat using seat pad made by Endevco Corporation (for location of the accelerometers see Figure 8).

The results obtained with the vibrating platform and the person who participated in this test with the automotive seat used showed that the seat dynamic behavior on the range from 4Hz to 400Hz frequency does not protect the user in regard to comfort (as can be seen in Table 2 [3, 8]).

In addition, specifically in the frequency range from 8Hz to 12Hz the spine shows greater sensitivity to exposure to harmful levels of vibration. In this frequency range various parts of the human body come into resonance. Tests have shown that in this case the seat did not protect the user for this specific range, so the subject was exposed to levels of inadequate bands of acceleration.

The system developed allows distinguishing human vibrations of almost 400Hz in three axes with acceleration of almost 50g. The receiver board developed can be attached to a PC through the RS-232 or USB interface allowing the reception of data continuously, if wanted (the limit of hours, days, weeks or months is related to the computer used and space available to store the data file). The tests conducted ensured the correct functioning of the system for the proposal of this project.

## Figures and Tables

**Table 1.** Average results.

Input Signal	Acceleration on the base (m/s^2^) (accelerometer 2)	Acceleration on the seat (m/s^2^) (seat pad – accelerometer 1)
4Hz	1.5	1.7
30Hz	2.6	3.4

**Table 2.** Global pondered acceleration and reactions indication regarding comfort.

Acceleration rms (m/s^2^)	Reaction
< 0.315	Comfortable
0.315 to 0.63	Slightly uncomfortable
0.8 to 1.6	Uncomfortable
1.25 to 2.5	Very uncomfortable
> 2.0	Extremely uncomfortable

## References

[1] Bao M.H. (2000). Micro mechanical transducers: pressure sensors, accelerometers and gyroscopes (Handbook of Sensors and Actuators).

[2] Balbinot A., Brusamarello V. (2007). Instrumentação e Fundamentos de Medidas – Volumes I e II.

[3] Griffin M.J. (1990). Handbook of human vibration.

[4] Johanning E., Wilder D.G., Landrigan P.J., Pope M.H. (1991). Whole-body Vibration Exposure in Subway Cars and Review of Adverse Health Effects. J. Occup. Med..

[5] Hoy J., Murabarak N. (2000). The Effect of Whole-body Vibration on Forklift Drivers.

[6] Tripepi M.G., Cantio M., Saffioti G. (2000). Risk and Effects of WBV in Locomotive Engineers.

[7] Hulshof C.T.J., Braam I.T.J., Verbeek J. (2000). Criteria for Recognition of Whole-body Vibration Injury as Occupational Disease: A Review.

[8] ISO 2631-1 (1997). Mechanical Vibration and Shock – Evaluation of Human Exposure to Whole-body Vibration – Part I: General Requirements.

[9] Griffin M.J. (1990). Effects of Horizontal Whole-body Vibration on Reading Applied Ergonomics. Ergonomics.

[10] Ishitake T., Matoba T. (2000). Frequency Weighting for the Effects of Exposure to Whole-body Vibration on Gastric Motility.

[11] Bongers P.M., Boshuizen H.C., Hulshof C.T.J., Koemeester A.P. (1988). Back Disorders in Crane Operators Exposed to Whole-Body Vibration. Int. Arch. Occup. Environ. Health.

[12] Palmer K.T., Griffin M.J., Bendall H., Pannett B. (2000). Prevalence and Pattern of Occupational Exposure to Whole Body Vibration in Great Britain: Findings from a National Survey. Occupational Environmental Medicine.

[13] Balbinot A., Bagesteiro L., Tamagna A. (2000). A Preliminary Study of the Drivers/Seat Interface to Driver´s Shoulder Transmissibility on Urban Buses in Porto Alegre-Brazil.

[14] Balbinot A., Brusamarello V., Gertz L.C., Laranja R.C. (2004). Vibração e Temperatura Localizada: sua relação com a coluna vertebral. IV Congresso Internacional de Automação, Sistemas e Instrumentação - ISA South América.

[15] IEEE Wireless Medium Access Control (MAC) and Physical Layer (PHY) Specifications for Low-Rate Wireless Personal Area Networks (LR-WPANS) (2003). IEEE standard 802.15.4.

